# Texas State Medical Association—Program

**Published:** 1894-04

**Authors:** H. A. West, A. N. Denton

**Affiliations:** Galveston, Secretary; Chairman Arrangement, Austin


### Society Notes.

### TEXAS STATE MEDICAL ASSOCIATION.

### Preliminary Announcement and Program of the Twenty-Sixth Annual Meeting, to be Held in the Senate Chamber, Capitol Building, Austin, Texas, April 24th, 25th, 26th, and 27th, 1894.

DEAR DOCTOR:—You are cordially invited to be present at the next annual meeting of the Texas State Medical Association, which will convene at the city of Austin, on the 24th day of April inst. We expect to have a brotherly and harmonious gathering of the best representatives of the medical profession from every section of this great State. An important matter for discussion will be the Code of Ethics of the American Medical Association, to determine whether we shall recommend any change to meet present advancement. We invite every medical gentleman belonging to the regular profession, to unite with us in one strong brotherhood to battle for the commonwealth against charlatans, imposters and mountebanks of every description, and to contend for the rights of our people, for protection by proper laws—both State and National—against all preventable epidemics, and for a cabinet position of health in general government and State. Yours truly,

J. H. SEARS, President.

#### TRANSPORTATION.

All the railroads in the State have agreed on conditions named below, *and will make a rate of one and one-third fare for the round trip*, on the certificate plan.

Certificates must be stamped by P. J. Lawless, ticket agent at Austin, and must be signed by Dr. H. A. West, secretary, as directed herein. On the certificate plan, the passenger pays full fare going to the meeting, and secures a receipt therefor, from the ticket agent, by request, at the time of purchase. This receipt is a certificate, which, when stamped by the ticket agent at place of meeting, and filled out and signed by the proper official at the meeting, becomes authority for the sale of a return ticket over the same route as was used on the going trip, at one-third fare, thus making a rate of one and one-third fare for the round trip.

When the journey is made over more than one line, it is frequently necessary for the passenger to purchase a separate local ticket and procure a receipt and certificate therefor from each of the lines over which he travels in going to the meeting. Passengers should therefore ascertain what portion of the journey can be made by the ticket procured from each agent, and should purchase tickets and secure certificates as may be necessary. Failure to procure or present a certificate invalidates any claim for reduction in return fare.

The ticket purchased for going passage may be either unlimited or limited, according to the rate paid, or regulations in effect on the line over which it reads, but the return ticket sold at the reduced fare, will, in all cases, be limited to continuous passage, beginning at the date of issue. Certificates will not be honored for return tickets at reduced rates unless presented on last day of meeting, or not later than one day after adjournment; nor will the certificates be honored if the receipts attached show that going tickets were purchased more than two days prior to the commencement of the meeting, or after the close thereof.

### HOTEL RATES.

Hotels will charge the following rates: Driskill, $2.50; Hotel Salge, $2.00; Avenue, $2.00; Orr, $1.50; Austin House, $1.25.

The above rates conditioned upon two or more occupying a room. Dr. Q. C. Smith, 617 Colorado street, Austin, will take pleasure in securing, in advance, rooms at hotels or private boarding houses for those who so request.

### PRELIMINARY PROGRAM.

The session will begin Tuesday morning at 11 o'clock, in the senate chamber of the capitol building. Members are requested to assemble early in order to register, as far as possible, before the opening. The Secretary, Treasurer and Members of the Reception Committee will be present to attend to registration. Applicants for membership will sign blank form, giving postoffice address and county. Each application must have the signatures of two members, and be accompanied with the initiation fee, $5.00, dues for one year, $5.00, and the diploma or other evidence of graduation in a regular medical school. Applicants will receive badges when they exhibit the Treasurer's receipt.

Members will receive badges after they have registered.

As the titles of papers appearing in this program were received by the Secretary in time to be arranged in their proper places, they will take precedence over those offered subsequently.

Morning session, after Tuesday, will begin at 9 o'clock. Afternoon session at 2:30 o'clock.

SOCIAL PROGRAM.

Wednesday evening at 8 o'clock—President's annual address, Dr. J. H. Sears, Waco, to be followed by the annual oration, "The Physician," Dr. W. R. Blailock, McGregor.

Thursday evening at 9 o'clock—Buffet banquet and reception at the Driskill.

Friday, 3:30 p. m.—Visit to the dam and excursion up the lake on the "Ben Hur," starting from corner Congress Avenue and Fourth street.

SECTION ON PRACTICE OF MEDICINE.

1. Report of Chairman, J. C. Jones, M. D., Gonzales.
2. *Bacteriological Aspects of Croupous Pneumonia.* H. A. West, M. D., Galveston.
3. *The Therapeutic Uses of Sparteine.* David Cerna, M. D., Ph. D., Galveston.
4. *Hydrotherapy.* D. R. Wallace, M. D., LL. D., Waco.
5. *The Relation of Pharmacy to Medicine.* James Kennedy, M. D., Galveston.
6. *Introducing the Subjects of Dosimetry and Subcutaneous Medication.* D. R. Wallace, M. D., LL. D., Waco.

SECTION ON OBSTETRICS AND DISEASES OF CHILDREN.

1. Report of Chairman, Edward Randall, M. D., Galveston.
2. *Symphysiotomy, and its Relation to other Obstetrical Operations.* J. F. Y. Paine, M. D., Galveston.
3. *Report of a Monstrosity Without Limbs or Sexual Organs.* Matthew M. Smith, B. Sc. M. A., M. D., Austin.

4. Paper (title not given). L. L. Jones, M. D., Forney Texas.

SECTION ON SURGERY.

1. Report of Chairman, C. A. Smith, M. D., Tyler.
2. *Tuberculosis of the Joints, its Etiology and Treatment.* Wm. M. Cunningham, M. D., of Bastrop. Discussed by Drs. W. F. Starley, of Tyler, and I. E. Clark of Schulenberg.
3. *Diseased Joints in Children.* Samuel E. Milliken, M. D., New York.
4. *The Treatment of Neglected Cases of Hip Joint Disease.* J. E. Thompson, M. D., Galveston.
5. *Removal of Female Breast and Axillary Glands for Malignant Disease.* B. F. Britain, M. D., of Arlington.
6. *A Report of Six Cases of Depressed Fracture of Frontal Region of Skull, with Two Deaths.* J. S. Price, M. D., of Beaumont. Discussed by Drs. A. F. Sampson, Galveston, and Matthew M. Smith, M. D., Austin.
7. *Brain Surgery of To-Day.* Matthew M. Smith, M. D., Austin.
8. *Concussion and Contusion of the Brain.* T. F. Wynn, M. D., Pittsburg.
9. *A Contribution to the Study of the Action of Chloroform.* Drs. Edward Randall and David Cerna, Galveston.
10. *Chloroform Anesthesia.* Q. C. Smith, Austin. Discussed by Drs. H. K. Leake, Dallas, and H. C. Nott, Goliad.
11. *Irrigation and Drainage for Peritonitis.* Joseph Price, M. D., Philadelphia, Pa. Discussed by Drs. B. E. Hadra, San Antonio, and C. A. Smith, Tyler.
12. *The Modern Treatment of Wounds.* Russell A. Hibbs, New York City. Discussed by Drs. T. J. Bennett, Austin, and George W. Christian, Houston.
13. *Report of a Case of Injury to the Chest.* August Schenk, M. D., Kenney.
14. *Appendicitis; also Report of Case of Congenital Displaced Enlarged Liver, Mistakên for Tumor; Operation.* J. E. Gilchrest, M. D. Gainesville. Discussed by Drs. B. E. Hadra, San Antonio, and A. Sims, Weatherford.
15. *The Operative and Mechanical Treatment of Club-Foot, with Report of Cases.* T. W. Shearer, M. D., Wallisville.

16. *A Case of Incomplete Dislocation of the Head of Tibia Inwards, with Complete Outward Vertical Luxation of the Patella.* William J. Bever, M. D., Creek.
17. *On Perindorrhaphy by Duke's Method.* Thos. More Madden, M. D., Honorary Member, Dublin, Ireland.
18. *Abscess of Spleen in Child Three Months Old, Recovery.* W. A. Watkins, M. D., Kemp. Discussed by Drs. Q. C. Smith, Austin, and J. D. Burch, Aurora.
19. *The Fitness of the Climate of Texas for Operative Surgery, Demonstrated by Results in Recent Capital Cases,* by Drs. Beall, Walker and Capps, Fort Worth.

SECTION ON MEDICAL JURISPRUDENCE, ETC.

1. Report of Chairman, B. F. Brittain, M. D., Arlington.
2. *Morphine Suicides. Prevention. Treatment of Opium Poisoning.* E. D. Capps, Fort Worth.

SECTION ON STATE MEDICINE AND PUBLIC HYGIENE.

1. Report of Chairman, (*Progress of Preventive Medicine*). T. J. Bennett, M. D., Austin.
2. *Increase of Mental Unsoundness.* D. R. Wallace, M. D., LL. D., Waco. Discussed by F. S. White, M. D.
3. *A Comprehensive, Practical and Effective Health System Supervised by the State—An Imminent and Pressing Necessity,—To Procure Which the Texas State Medical Association, as the Legitimate Representative of the Profession of the State, Should Take the Initiative.* J. L. Cunningham, M. D., Fort Worth. Discussed by Drs. N. B. Kennedy, Hillsboro, and R. M. Swearingen, Austin.
4. *How to Prevent Typhoid Fever.* J. W. Carhart, M. D., Lampasas. Discussed by Drs. H. A. West, Galveston, and H. C. Black, Waco.
5. *Consumption, and How to Cure it.* W. R. Blailock, M. D., McGregor. Discussed by Drs. J. S. Lankford, San Antonio, and J. W. McLaughlin, Austin.
6. *Mind Cure.* D. R. Wallace, M. D., Waco.

SECTION ON GYNECOLOGY.

1. Report of Chairman, Geo. W. Christian, M. D.
2. *Dysmenorrhœa, with Sterility, and its Treatment.* Sam Cunningham, M. D., Elgin. Discussed by Drs. Hibbs, of New York, and G. B. Foscue, of Waco.

3. *An Original Method of Disposing of the Ligatures after Laparotomies in the Pelvic Region, Effecting Perfect Vaginal Drainage; With Report of Cases.* Joseph Cummings, of Austin. Discussed by Drs. W. J. Mathews, of Austin, and H. H. Thorppe, of Liberty Hill.
4. *Intra-Uterine Manipulations in Pelvic Inflammation.* J. F. Y. Paine, M. D., Galveston.
5. ]Paper (title not given). H. K. Leake, M. D., Dallas.
6. *Placenta Prævia; Treated by Cæsarian Section.* G. A. Moses, M. D., St. Louis, Mo. Discussed by Drs. T. D. Wooten, Austin, and George Cupples, San Antonio.
7. *The After Treatment of Cæiliotomies.* G. D. Parker, M. D., of Houston. Discussed by Drs. A. C. Scott, of Temple, and Q. C. Smith, of Austin.

SECTION ON DERMATOLOGY.

1. Report of Chairman, F. E. Daniel, M. D., Austin.
2. *The Use and Abuse of Arsenic in Skin Diseases.* Isadore Dyer, M. D., Tulane University, N. O.

SECTION ON MICROSCOPY AND PATHOLOGY.

1. Report of Chairman, Allen J. Smith, M. D., Galveston.
2. *Some Clinical Observations on Hæmatozoa Malariæ.* Wm. Gammon, M. D., Galveston.

So far no papers have been reported to the Secretary for the Section on Opthalmology.

H. A. WEST, M. D.,
Galveston, Secretary.

A. N. DENTON, M. D.,

Chairman Arrangement, Austin.

---

To Microscopists.—Prof. Allen J. Smith, Professor Pathology, etc., in Medical Department University Texas, and Chairman of Section on Microscopy and Pathology Texas State Medical Association, requests the JOURNAL to say that he will be pleased to correspond with any physician who has matter of interest in microscopy or pathology to announce; and that all such persons are invited to contribute same to his Section for the April (24 to 28) meeting of State Medical Association at Austin.

